# Polymeric Therapeutic Nanosystems Containing Paclitaxel: Novel Strategies, Therapeutic Potential, Challenges, and Translation Problems

**DOI:** 10.3390/ma19142999

**Published:** 2026-07-11

**Authors:** Marcin Sobczak, Karolina Kędra

**Affiliations:** 1 Department of Pharmaceutical Chemistry and Biomaterials, Faculty of Pharmacy, Medical University of Warsaw, Banacha 1 Str., 02-097 Warsaw, Poland; 2 Institute of Physical Chemistry, Polish Academy of Sciences, 44/52 Kasprzaka Str., 01-224 Warsaw, Poland; kkedra@ichf.edu.pl

**Keywords:** paclitaxel, biomedical polymers, polymeric drug delivery systems, nanocariers, cancer therapy, targeted delivery

## Abstract

Cancers still remain one of the most significant challenges in medicine or pharmacy, accounting for nearly 10 million deaths annually and imposing a substantial socioeconomic burden worldwide. Although chemotherapy continues to play a central role in the treatment of many tumors, conventional anticancer therapies are frequently associated with poor selectivity, systemic toxicity, multidrug resistance, and unfavorable pharmacokinetic profiles. Paclitaxel (PTX), one of the most widely used antineoplastic agents, demonstrates remarkable clinical efficacy against breast, ovarian, lung, pancreatic, and several other malignancies. Nevertheless, its clinical application remains limited by poor aqueous solubility, non-specific biodistribution, dose-limiting toxicities, and the development of resistance mechanisms. Nanotechnology-based anticancer drug delivery systems have emerged as a promising strategy to address these limitations. Among them, polymeric nanosystems have attracted particular attention owing to their physicochemical properties, biocompatibility, controlled drug-release capabilities, and potential for tumor-targeted delivery. Natural, semi-synthetic, and synthetic polymers are extensively investigated as carriers for PTX, leading to the development of nanoparticles, micelles, nanogels, nanofibers, dendritic systems, and hybrid nanoplatforms. Nanosystems demonstrate enhanced therapeutic efficacy, reduced systemic toxicity, prolonged circulation times, and improved tumor accumulation in preclinical models. Despite encouraging laboratory results, the clinical translation of polymeric PTX nanocarriers (NCs) remains limited. Numerous barriers, including tumor heterogeneity, variability of the enhanced permeability and retention (EPR) effect, manufacturing complexity, regulatory challenges, scale-up difficulties, and discrepancies between animal models and human cancers, continue to hinder successful commercialization and widespread clinical adoption. This review critically discusses the current state of polymeric drug delivery systems (DDSs) that contain PTX, as well as the advantages and limitations of synthetic, natural, and semi-synthetic polymers used in DDS technologies. Furthermore, translational challenges and future perspectives of PTX-based DDSs were analyzed.

## 1. Introduction

Cancer continues to represent one of the most serious public health problems worldwide. According to the World Health Organization (WHO), approximately 20 million new cancer cases and nearly 10 million cancer-related deaths were reported globally in 2022, with projections indicating a substantial increase in incidence over the next two decades [1,2,3]. Population aging, urbanization, environmental pollution, obesity, sedentary lifestyles, and tobacco consumption are among the major contributors to this growing burden. Despite considerable advances in molecular diagnostics, surgical techniques, immunotherapy, radiotherapy, and targeted therapeutics, chemotherapy remains an indispensable component of cancer treatment [4,5]. However, conventional chemotherapy suffers from several inherent limitations. Most anticancer drugs exhibit poor selectivity toward malignant tissues and therefore damage rapidly dividing healthy cells. Consequently, severe adverse effects such as myelosuppression, gastrointestinal toxicity, mucositis, alopecia, and immunosuppression frequently occur [6]. Furthermore, the emergence of multidrug resistance (MDR) significantly compromises therapeutic efficacy and remains one of the leading causes of treatment failure and disease recurrence [2].

One approach to solving the above problems is nanotechnology-based DDSs. Nanomedicine utilizes nanoscale materials and nanostructures to enhance drug solubility, pharmacokinetics, biodistribution, therapeutic efficacy, and safety profiles. Since the introduction of the first clinically approved nanomedicines, numerous NC platforms have been investigated, including liposomes, polymeric nanoparticles (NPs), micelles, dendrimers, nanogels, metallic NPs, carbon nanotubes, and hybrid systems [7,8]. The unique physicochemical characteristics of NCs, including their nanoscale dimensions, large surface-area-to-volume ratio, modifiable surface chemistry, and ability to encapsulate hydrophobic drugs, enable the development of sophisticated delivery platforms. NCs can prolong systemic circulation, protect encapsulated drugs from premature degradation, facilitate controlled release, and enhance accumulation at tumor sites [4]. Historically, tumor accumulation of NPs was largely attributed to the EPR effect, whereby defective tumor vasculature and impaired lymphatic drainage facilitate passive nanoparticle (NP) extravasation. However, recent evidence suggests that the EPR effect may be significantly less pronounced and more heterogeneous in human tumors than originally believed. Consequently, many nanoparticle formulations demonstrating remarkable efficacy in murine models have failed to reproduce similar outcomes in clinical studies [6,9]. Among currently available chemotherapeutic agents, PTX is among the most extensively investigated candidates for nanotechnology-based DDSs. Owing to its potent antimitotic activity, broad-spectrum antitumor efficacy, and well-established clinical role, PTX has become a model drug for evaluating novel nanocarrier (NC) systems [1,10,11]. It was found that PTX-loaded nano drug delivery systems (nano-DDSs) demonstrate substantial improvements in solubility, pharmacokinetics, tumor targeting, and toxicity profiles. Polymeric NCs, in particular, have attracted considerable attention due to their structural versatility, biocompatibility, biodegradability, and the ability to be functionalized with targeting ligands, imaging agents, and stimuli-responsive components [12,13].

Although numerous review articles on PTX-based nanomedicine have been published in recent years, most have focused on relatively narrow aspects of PTX delivery systems. Recent reviews have focused primarily on individual nanocarrier platforms, such as electrospun nanofibers, polymeric NPs with active targeting ligands, or co-delivery systems developed for a single cancer type, particularly breast cancer. Consequently, these reviews provide valuable but highly specialized perspectives that do not fully reflect the current diversity of polymeric drug delivery strategies or the complexity of their clinical translation. Moreover, the rapidly expanding number of newly developed polymeric formulations, together with growing evidence that questions the predictive value of conventional preclinical models, necessitates an updated, more comprehensive critical evaluation of the field.

The present review aims to fill this gap by providing an integrated analysis of polymeric therapeutic nanosystems containing PTX across the entire spectrum of currently investigated polymeric carriers, including synthetic, natural, and semi-synthetic polymers as well as NPs, polymeric micelles, nanogels, nanofibers, dendritic systems, polymer-drug conjugates, and hybrid nanoplatforms. Unlike previous reviews, we critically compare the advantages and limitations of individual polymer classes, discuss their influence on drug delivery performance and therapeutic efficacy, and, importantly, evaluate the major barriers that continue to impede successful clinical translation. Particular emphasis is placed on tumor heterogeneity, the limited clinical relevance of the enhanced permeability and retention (EPR) effect, manufacturing and scale-up challenges, regulatory considerations, reproducibility, and the discrepancy between encouraging preclinical outcomes and the relatively small number of clinically successful polymeric PTX formulations. By integrating recent experimental evidence with clinically approved formulations and translational considerations, this review provides a broader perspective on the current state of polymeric PTX delivery systems and identifies the most promising directions for future research and clinical development.

## 2. Paclitaxel

**Figure 1:**
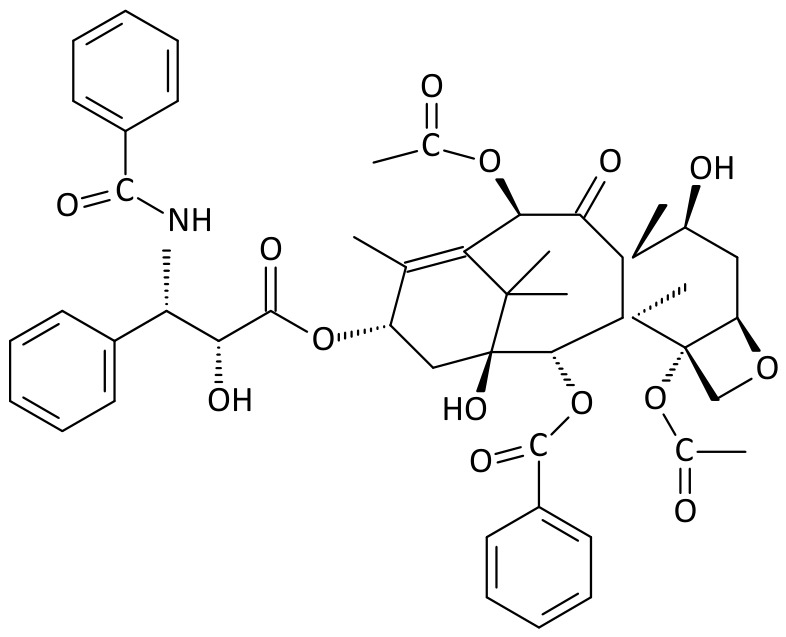
Structure of paclitaxel.

PTX was originally isolated from the bark of the Pacific yew tree (*Taxus brevifolia*) in the 1960s. The compound was structurally characterized in 1971 and subsequently emerged as one of the most successful natural-product-derived anticancer drugs (Figure 1). However, the main problem turned out to be the process of obtaining this active substance, which is expensive and requires a large amount of natural raw material. It has been estimated that several mature trees were necessary to produce only a few grams of PTX [1,10,14].

To address these limitations, alternative production strategies were developed, including semi-synthetic synthesis from 10-deacetylbaccatin III, plant cell culture technologies, endophytic fungal fermentation, total chemical synthesis, and metabolic engineering. Among these methods, semi-synthetic production remains the dominant industrial route due to its economic feasibility and scalability [1,10,14]. PTX belongs to the taxane family. The molecule of PTX contains a complex tetracyclic taxane ring system consisting of an eight-membered taxane core fused with a four-membered oxetane ring and an esterified side chain attached at the C13 position. The side chain, N-benzoyl-(2R,3S)-3-phenylisoserine, is primarily responsible for the drug’s interaction with β-tubulin and is essential for its antitumor activity [1,6,10,14].

PTX exhibits highly unfavorable physicochemical characteristics. The compound is extremely hydrophobic, displaying aqueous solubility below 1 μg/mL under physiological conditions. Its logP value exceeds 3.5, reflecting pronounced lipophilicity and poor dissolution in biological fluids. These properties substantially limit bioavailability and necessitate formulation strategies that enhance solubility and systemic delivery [1,6]. As is well known, PTX’s poor aqueous solubility has been a major obstacle to its pharmaceutical development. Traditional intravenous formulations require the use of Cremophor EL^®^ (polyoxyethylated castor oil) and ethanol as solubilizing agents. However, these excipients contribute significantly to formulation-related toxicity, hypersensitivity reactions, complement activation, and altered pharmacokinetics [6,10]. The physicochemical limitations of PTX have therefore become a principal driving force behind the development of nano-DDSs. Polymeric NPs, micelles, nanogels, dendrimers, and nanofibers containing PTX have been specifically engineered to address these challenges by improving aqueous dispersibility and stability, enabling controlled release, and reducing solvent-associated toxicities [12,15].

## 3. Formulations of Paclitaxel

### 3.1. Formulations Without Polymer Components

The clinical success of PTX has been accompanied by persistent pharmaceutical challenges related to its extremely poor aqueous solubility. Since PTX is practically insoluble in water, the development of clinically viable formulations has required specialized excipients and DDSs. Over the past three decades, formulation strategies have evolved from solvent-based systems to albumin-bound NPs, polymeric micelles, and advanced nanotechnology platforms aimed at improving the therapeutic index while minimizing systemic toxicity [6,10]. Initially, formulation efforts focused primarily on improving solubility. More recently, however, emphasis has shifted toward controlling biodistribution, reducing off-target toxicity, overcoming multidrug resistance, and enabling tumor-selective delivery through nano-DDSs [6,12].

Taxol^®^ was the first PTX formulation approved by the United States Food and Drug Administration (FDA) in 1992 for the treatment of ovarian cancer. Subsequent approvals expanded its indications to include breast cancer, non-small-cell lung cancer, Kaposi’s sarcoma, and several other malignancies [1,6]. To overcome PTX insolubility, Taxol^®^ utilizes a mixture of Cremophor EL^®^ and ethanol as solubilizing agents. While this approach successfully enables intravenous administration, it introduces numerous formulation-associated toxicities that significantly limit treatment tolerability. Cremophor EL has been implicated in severe hypersensitivity reactions, peripheral neuropathy, altered drug distribution, nonlinear pharmacokinetics, nephrotoxicity, and vascular irritation. An important limitation of Taxol^®^ is its relatively poor tumor selectivity. Following administration, substantial drug accumulation occurs in healthy tissues, contributing to dose-limiting toxicities. These adverse effects often necessitate dose reductions or discontinuation of treatment. Although Taxol^®^ remains widely used worldwide, its shortcomings have stimulated intensive research aimed at developing safer and more effective formulations [1,6,7,10].

A breakthrough in PTX formulation occurred with the development of Abraxane^®^ (nab-paclitaxel, nab-PTX), an albumin-bound nanoparticle formulation approved by the FDA in 2005 [6,10]. Abraxane^®^ consists of approximately 130 nm NPs composed of PTX non-covalently associated with human serum albumin. This formulation eliminates the need for Cremophor EL and ethanol, thereby avoiding many solvent-related adverse reactions [6]. Several pharmacological advantages have been associated with Abraxane^®^: shorter infusion times, higher tolerated doses, improved drug delivery, and reduced solvent-associated toxicity. Clinical trials demonstrated improved response rates in metastatic breast cancer compared with conventional Taxol^®^. Similar benefits have been observed in pancreatic cancer and non-small cell lung cancer when nab-PTX is combined with standard chemotherapeutic regimens [1,6]. Despite these advantages, Abraxane^®^ is not without limitations. Manufacturing costs remain substantially higher than those of conventional formulations. Furthermore, the magnitude of pharmacokinetic improvement is often smaller than initially anticipated, and clinically significant neurotoxicity and myelosuppression remain important concerns [10].

### 3.2. Polymeric Nano-Drug Delivery Systems Containing Paclitaxel

An ideal PTX delivery system should increase aqueous solubility, eliminate toxic excipients, improve tumor accumulation, reduce exposure of healthy tissues, overcome multidrug resistance, enable controlled release, facilitate combination therapy, and improve patient quality of life. Polymeric nanosystems containing PTX offer the potential to address all these challenges simultaneously. Consequently, they have become one of the most extensively investigated drug-delivery platforms in contemporary oncology.

**Figure 2:**
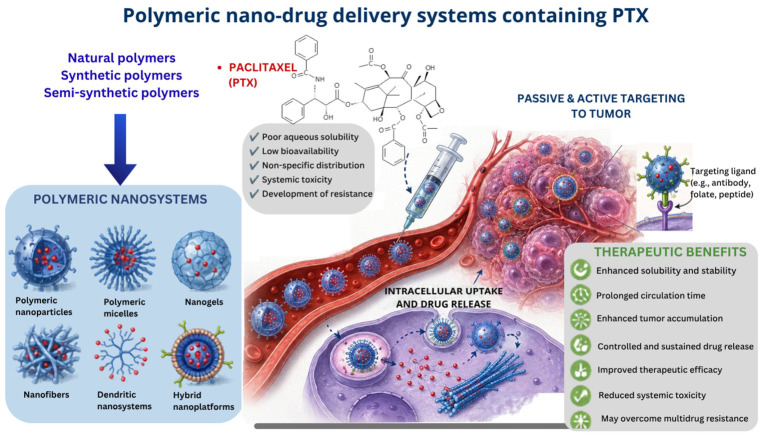
Types of therapeutic systems containing paclitaxel. Some graphic elements (syringe, cell, blood vessel) were generated by AI. The manuscript authors independently prepared the figure concept, scheme overall layout, Paclitaxel formula, descriptions, and other graphic elements using the graphic design software ChemWin, Canva, and Paint.

The success of polymeric NCs largely depends on the physicochemical and biological properties of the polymers used during formulation development. The ideal polymer for PTX delivery should be biocompatible, biodegradable, non-immunogenic, capable of achieving high drug loading, amenable to large-scale manufacturing, and suitable for controlled drug release. In practice, no single polymer fulfills all these requirements. Consequently, numerous synthetic, natural, and semi-synthetic polymers have been investigated individually or in combination to optimize PTX-DDSs (Figure 2, Table 1) [4,8,12,14]. The choice of polymer influences virtually every aspect of nano-DDS performance, including NP size, surface charge, circulation time, biodistribution, intracellular uptake, degradation kinetics, drug release behavior, and toxicity profile. Importantly, polymer selection also determines the likelihood of successful clinical translation because regulatory agencies generally favor materials with established safety records and scalable manufacturing processes [8,9,12]. Among currently investigated PTX nano-DDSs, synthetic polymers remain the most extensively studied group owing to their reproducibility, structural versatility, and ease of industrial production. Several synthetic polymers have already received regulatory approval for various biomedical applications, making them particularly attractive candidates for translational nanomedicine [8,12].

The rationale for encapsulating PTX in polymeric NCs stems from several major limitations of conventional PTX formulations. These include poor aqueous solubility, non-specific biodistribution, rapid clearance from systemic circulation, toxicity associated with formulation excipients, and the development of multidrug resistance. Polymeric carriers provide a versatile framework that can simultaneously address these challenges through careful engineering of size, surface chemistry, drug loading, and release kinetics [5,15,60,61].

Polymeric PTX DDSs can be broadly classified into polymeric NPs, polymeric micelles, nanogels, dendrimers, polymer-drug conjugates, nanofibers, and hybrid polymeric nanostructures. Each platform possesses distinct physicochemical characteristics that influence biodistribution, drug release, cellular uptake, and therapeutic efficacy [16,17,18,19,20,21,22,23,24,25,26,27,28,29,30,31,32,33,34,35,36,37,38,39,40,41,42,43,44,45,46,47,48,49,50,51,52,53,54,55,56,57,58,59,60,61].

#### 3.2.1. Synthetic Polymers

Synthetic polymers dominate the field of PTX-nano-DDSs technology (Table 1) [31,32,33,34,36,37,43,44,45,46,47,48,49,50,51,52,53,54,55,56,57,58]. Their major advantages include reproducible chemical composition, controlled molecular weight and distribution, tunable degradation kinetics, scalable manufacturing, and straightforward surface modification. However, synthetic polymers may also exhibit limitations, including insufficient biological recognition, potential accumulation of degradation products, and relatively limited intrinsic bioactivity compared with natural polymers [8,12,14].

**Figure 3:**
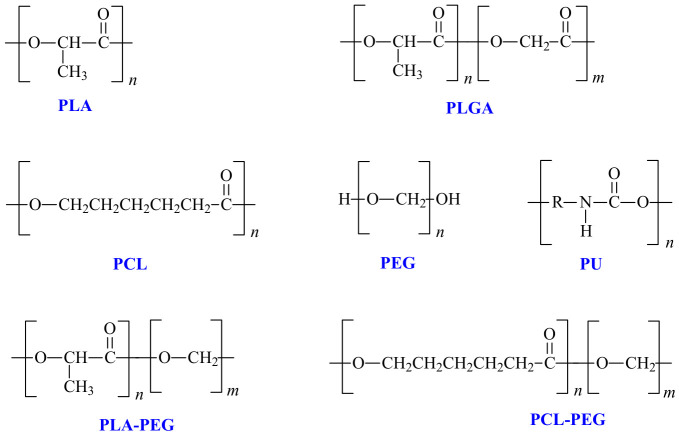
The most commonly used synthetic polymers in paclitaxel nanosystems technology.

The most important synthetic polymers used in PTX nano-DDSs include poly(lactic-co-glycolic acid) (PLGA), poly(lactic acid) (PLA), polycaprolactone (PCL), polyethylene glycol (PEG), PEG-containing block copolymers, poly(amino acid)-based polymers, polyurethanes (PU), and poly(amidoamine) dendrimers (Figure 3) [31,32,33,34,36,37,43,44,45,46,47,48,49,50,51,52,53,54,55,56,57,58].

**Figure 4:**
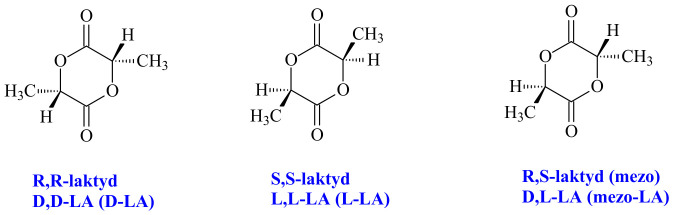
Stereoisomers of lactide.

PLA is a biodegradable polyester derived from lactic acid. It may be synthesized through direct polycondensation of lactic acid or ring-opening polymerization of lactide (LA). LA (dimer of lactic acid) has two chiral centers (Figure 4). Consequently, it can exist as three stereoisomers (Figure 4).

The polymer exists in several stereochemical forms: poly(L-lactide) (PLLA), poly(D-lactide) (PDLA) and poly(D,L-lactide) (PDLLA) [43,44,45]. Differences in stereochemistry strongly influence crystallinity, biodegradation, and the kinetics of drug release. PLA exhibits high hydrophobicity, good mechanical strength, prolonged degradation times, and favorable biocompatibility. These properties enable efficient loading of hydrophobic PTX. However, the relatively slow degradation of PLA may result in prolonged persistence of NPs within tissues [4,8,43,44]. PTX-loaded PLA NPs demonstrate improved drug stability, prolonged release, enhanced tumor suppression, and reduced systemic toxicity. PTX-based PLA-DDSs frequently demonstrate excellent preclinical performance. However, their long half-lives may complicate clinical translation when repeated dosing is required. Consequently, many modern formulations increasingly employ PLA in combination with PEG or other polymers to balance stability and biodegradation [14,15,43,44].

PLGA is a biodegradable aliphatic polyester composed of lactic acid and glycolic acid monomer units. The polymer is typically synthesized through ring-opening polymerization of LA and glycolide (GA) under controlled catalytic conditions [48,49,50,51,52,53,54,55]. The LA:GA ratio strongly influences degradation kinetics. Increasing GA content generally accelerates hydrolysis due to enhanced hydrophilicity [4,8,14,53,54]. PLGA degradation occurs primarily through hydrolytic cleavage of ester bonds, yielding lactic acid and glycolic acid, which subsequently enter endogenous metabolic pathways, including the Krebs cycle. PLGA exhibits several properties favorable for PTX delivery: biocompatibility, biodegradability, high drug encapsulation efficiency, sustained drug release, and ease of nanoparticle fabrication. NPs prepared from PLGA typically range from 80 to 300 nm in diameter, depending on formulation conditions [12,14,53,54,56]. The hydrophobic nature of PLGA facilitates efficient incorporation of PTX into the polymer matrix. PLGA remains one of the most widely investigated NCs for PTX. PLGA NPs generally demonstrate improved PTX solubility, prolonged circulation, controlled drug release, and reduced systemic toxicity. Numerous studies have reported significantly greater antitumor efficacy than free PTX in breast, lung, ovarian, and pancreatic cancer models [8,9,12]. PLGA NPs can be functionalized with folic acid, hyaluronic acid, transferrin, antibodies, and aptamers. Such systems enhance receptor-mediated uptake and often improve intracellular PTX accumulation in resistant tumors [9,12]. PLGA offers several important advantages: excellent safety profile, regulatory acceptance, controlled degradation, high drug-loading efficiency, and ease of formulation. Despite its strengths, PLGA presents several challenges. The acidic degradation products generated during hydrolysis may destabilize encapsulated therapeutics, induce local inflammation, and accelerate PTX degradation. Furthermore, PTX release kinetics are often characterized by an undesirable initial burst release that may compromise long-term control [8,9,12,14,56].

PCL is a semicrystalline polyester produced by ring-opening polymerization of ε-caprolactone (CL) [37,38]. Compared with PLGA and PLA, PCL exhibits greater hydrophobicity, slower degradation, and excellent mechanical properties. These characteristics make PCL particularly attractive for long-term controlled-release systems. PTX-loaded PCL NPs demonstrate sustained release lasting weeks to months, enhanced stability, and high encapsulation efficiency. PCL is frequently combined with PEG to produce PEG-PCL micelles, one of the most extensively investigated platforms for PTX. The prolonged PTX release provided by PCL may be advantageous in localized delivery systems but less desirable for systemic chemotherapy, where precise pharmacokinetic control is required [14,15,56].

As is known, PEG is one of the most widely utilized polymers in pharmaceutical technology, biotechnology, and nanomedicine due to its excellent aqueous solubility, biocompatibility, and low toxicity [12,15,34,35,36]. Unlike PLGA, PLA, or PCL, PEG is generally not used as the primary structural component of PTX NPs. Instead, it serves as a surface-modifying polymer or as the hydrophilic block of amphiphilic copolymers used to construct polymeric micelles and hybrid nanosystems [15]. PEG is incorporated into numerous PTX-nano-DDSs, including PEG-PLA micelles, PEG-PCL micelles, PEG-PLGA NPs, PEGylated dendrimers, polymer-drug conjugates, and hybrid nanosystems. Among these, PEG-containing polymeric micelles remain particularly important because they effectively solubilize hydrophobic PTX without the need for Cremophor EL. The hydrophobic polymer block forms the micellar core, encapsulating PTX, whereas PEG forms the hydrophilic corona, which stabilizes the micelles in aqueous environments. This architecture provides several therapeutic advantages, including improved solubility, reduced aggregation, prolonged circulation, enhanced tumor exposure, and improved pharmacokinetic profiles [15,34,40].

PEG–PLGA block copolymers are among the most extensively investigated nano-DDSs for PTX. The amphiphilic nature of PEG–PLGA allows spontaneous self-assembly into polymeric micelles, NPs, nanocapsules, and hybrid nanostructures. These systems can efficiently encapsulate hydrophobic PTX molecules within the PLGA core while PEG forms a hydrophilic corona [12,15,36,53]. When dispersed in aqueous media above the critical micelle concentration (CMC), these copolymers spontaneously organize into nanoscale assemblies [15,53,54]. The resulting nanostructures typically range from 20 to 150 nm and exhibit excellent colloidal stability. PTX loading efficiencies commonly exceed 70–90% encapsulation efficiency and 5–20% drug-loading capacity (depending on formulation conditions) [12,56]. Compared with conventional Taxol^®^, PEG–PLGA DDSs offer substantially enhanced aqueous dispersibility, elimination of Cremophor EL, improved pharmacokinetic profiles, and controlled release. Importantly, release kinetics can be adjusted by modifying PEG and PLGA molecular weight, PLGA microstructure, and the LA:GA ratio. PEG–PLGA NPs generally demonstrate prolonged systemic circulation owing to PEG-mediated reduction in protein adsorption and macrophage recognition. Several studies have reported increased plasma half-life, reduced hepatic clearance, improved tumor accumulation, and enhanced intratumoral retention [12,36,53,56]. PEG–PLGA NPs can be readily functionalized with targeting ligands. Frequently investigated modifications include folic acid (targets folate receptors overexpressed in ovarian cancer, breast cancer, lung cancer), hyaluronic acid (targets CD44 receptors present on breast cancer stem cells, ovarian cancer cells, pancreatic cancer cells), transferrin (targets transferrin receptors associated with rapidly proliferating tumors), and antibodies (including trastuzumab, cetuximab, anti-EGFR antibodies). These modifications frequently improve cellular uptake and therapeutic efficacy in preclinical models [12]. Although PEG–PLGA DDSs often outperform free PTX in animal models, clinical benefits are frequently more modest than anticipated. The emergence of polymeric nanotechnology has fundamentally transformed the field of anticancer drug delivery. Unlike conventional formulations, polymeric nanosystems can be engineered to control drug release, enhance pharmacokinetic behavior, improve tumor accumulation, reduce systemic toxicity, and facilitate active targeting of malignant tissues. Owing to these advantages, polymer-based delivery systems have become among the most extensively investigated platforms for PTX administration [4,12,40].

PEG–PCL copolymers combine hydrophilic PEG with hydrophobic PCL, as in PEG–PLGA, and these amphiphilic copolymers spontaneously self-assemble into polymeric micelles capable of encapsulating hydrophobic PTX [15,47]. The major distinction between PEG–PCL and PEG–PLGA systems arises from the slower degradation of PCL. PCL exhibits high crystallinity, low water permeability, and prolonged biodegradation times. Consequently, PEG–PCL micelles often demonstrate greater structural stability. PEG–PCL systems have been extensively investigated for PTX delivery. Many studies report enhanced tumor suppression, improved pharmacokinetics, and reduced systemic toxicity. PEG–PCL micelles often exhibit particle sizes of 20–80 nm, which is favorable for systemic administration. One particularly active area of research involves stimuli-responsive PEG–PCL formulations (pH-, redox-, or thermoresponsive). Such approaches seek to improve tumor selectivity and minimize off-target exposure. PEG–PCL micelles often outperform PEG–PLGA systems in terms of physical stability. However, superior physicochemical performance does not automatically translate into superior clinical outcomes [4,15,47].

Poly(amidoamine) (PAMAM), mainly as dendrimers, is a highly branched, monodisperse macromolecule with a central core, repetitive branching layers, and numerous terminal groups.

Each successive generation increases molecular size, surface functionality, and drug-loading potential. PTX can be incorporated via encapsulation, drug molecules occupy internal dendritic cavities, and covalent conjugation. Dendrimers are among the most sophisticated platforms in nanomedicine. However, increasing structural sophistication often comes at the cost of translational feasibility. From a clinical perspective, a slightly less effective but manufacturable and affordable nanoparticle may ultimately prove more successful than an extraordinarily complex dendritic system [2,12].

Poly(amino acid)-based polymers represent an important class of biodegradable synthetic biomaterials. Common examples include poly(aspartic acid), poly(glutamic acid), poly(lysine), poly(histidine), and poly(amino acid) copolymers. These materials possess excellent biocompatibility and offer opportunities for sophisticated molecular engineering [20,42]. One of the best-known examples is poly(L-glutamic acid)-PTX conjugate (CT-2103; Opaxio). This system was designed to improve tumor accumulation and reduce systemic toxicity. Although early clinical studies demonstrated promising pharmacokinetic properties, definitive clinical superiority over standard therapies was not established [20,42].

#### 3.2.2. Natural and Semi-Synthetic Polymers

**Figure 5:**
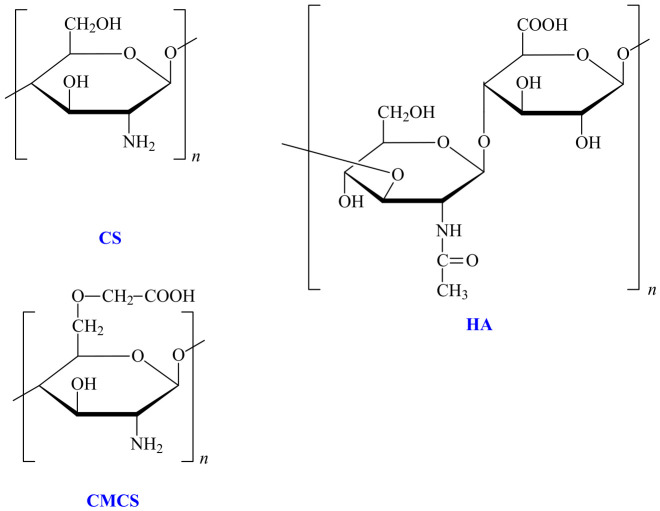
The most commonly used natural and semi-synthetic polymers in paclitaxel nanosystems technology.

Natural polymers have attracted considerable attention in the development of PTX-nano-DDSs because they possess intrinsic biological compatibility and biodegradability, and often exhibit bioactive properties that may enhance therapeutic performance (Table 1) [18,23,24,25,26,29]. Unlike synthetic polymers, many natural polymers contain functional groups that participate in biological interactions, facilitating cellular uptake, tissue adhesion, and receptor-mediated targeting [4,12,24,29]. However, natural polymers also present significant challenges, including batch-to-batch variability, structural heterogeneity, purification difficulties, limited mechanical stability, and scalability concerns. These factors have important implications for clinical translation and regulatory approval. Among the most important natural polymers investigated for PTX delivery are chitosan (CS), CS-derivatives (CMCS—carboxymethyl CS), hyaluronic acid (HA), alginate (ALG), dextran, cellulose derivatives, and cyclodextrins (Figure 5) [9,25,29].

Semi-synthetic polymers constitute an important class of biomaterials that bridge the gap between purely synthetic and naturally occurring polymers [16,17,19,20,21,22,27,28,30,35,38,39,40,41,42]. These materials are generated by chemical modification of natural polymers to improve physicochemical properties, pharmaceutical performance, stability, and manufacturability, while retaining many of the biological advantages of natural biomacromolecules [19,30,39].

In the context of PTX delivery, semi-synthetic polymers have emerged as particularly attractive candidates because they often combine the biocompatibility of natural polymers, the reproducibility of synthetic materials, enhanced drug-loading capacity, improved stability, and controlled degradation. Semi-synthetic polymeric nanosystems have been developed to address several shortcomings of unmodified natural polymers, including poor aqueous solubility, limited mechanical stability, rapid enzymatic degradation, and insufficient control over drug release kinetics. The most important semi-synthetic polymers investigated for PTX delivery include PEGylated CS derivatives, carboxymethyl CS, hydrophobically modified CS, modified Dex, hydroxypropyl CE derivatives, CD derivatives, and HA–synthetic copolymers [16,28,35,42].

CS is a linear polysaccharide produced through partial deacetylation of chitin, the second most abundant natural biopolymer after cellulose [16,17,18,19]. One of the most important features of CS is the presence of amino groups that become protonated under acidic conditions. As a result, CS exhibits positive surface charge, mucoadhesive properties, permeation-enhancing activity, and strong interactions with negatively charged biological membranes. PTX-loaded CS systems include NPs, nanogels, micelles, nanofibers, and hybrid polymeric systems. Several studies have demonstrated enhanced cellular uptake, prolonged circulation, and improved antitumor activity. The positive charge of CS NPs promotes interaction with negatively charged cancer cell membranes, frequently resulting in enhanced intracellular accumulation of PTX [1,19,23,54]. Stearic acid-modified CS facilitates self-assembly into micellar systems. Several PTX-loaded formulations based on these derivatives have demonstrated superior therapeutic outcomes compared with unmodified CS systems. CS is among the most biologically active polymers used in nanomedicine. However, its natural origin creates reproducibility challenges that are generally less problematic than those encountered with synthetic polymers [1,54].

PEGylated CS is produced through covalent attachment of PEG chains to amino or hydroxyl groups present within the CS backbone [17]. The resulting copolymer combines bioadhesion, biodegradability, and cellular interactions (CS) with prolonged circulation and improved aqueous solubility (PEG). Various synthetic routes have been described, including carbodiimide-mediated coupling, reductive amination, and click chemistry approaches [1,12,23]. PEGylated CS derivatives readily self-assemble into NPs, micelles, and nanogels. PTX-loaded PEGylated CS systems frequently demonstrate improved colloidal stability, prolonged circulation, and enhanced tumor accumulation. Several studies have reported superior antitumor efficacy compared with conventional PTX formulations in breast and ovarian cancer models [23,54].

Carboxymethyl CS (CMCS) is obtained through the introduction of carboxymethyl groups into the CS backbone [16]. This modification dramatically improves water solubility, physiological stability, and formulation versatility. Unlike CS, CMCS remains soluble over a broader pH range, making it particularly attractive for systemic drug-delivery applications. CMCS has been utilized to fabricate NPs, micelles, nanogels, and polymer-drug conjugates. PTX-loaded CMCS systems frequently exhibit controlled release, improved bioavailability, and enhanced tumor suppression. Several formulations have additionally incorporated targeting ligands and imaging agents to create multifunctional nanocarriers. CMCS is often considered more suitable than native CS for systemic PTX delivery because of its superior aqueous behavior. Nevertheless, the benefits of chemical modification must be weighed against increased production complexity [15,16,17,23].

Hydrophobically modified CS (with hydrophobic moieties such as stearic acid, cholesterol, and fatty acids) facilitates spontaneous self-assembly into micelle-like nanostructures that efficiently encapsulate PTX. These systems often demonstrate exceptionally high PTX loading, prolonged release, enhanced cellular uptake, and improved antitumor efficacy [15,16,17,18].

HA is an important component of the extracellular matrix, connective tissues, synovial fluid, and skin [24,25,26,27,28]. HA has attracted enormous interest because it naturally binds to the CD44 receptor, which is overexpressed in numerous tumors (breast cancer, ovarian cancer, pancreatic cancer, lung cancer, and colorectal cancer. PTX-loaded HA-DDSs include NPs, nanogels, micelles, and polymer-drug conjugates. Numerous studies have demonstrated enhanced tumor accumulation, improved intracellular uptake, and reduced systemic toxicity. HA-mediated targeting frequently increases PTX delivery specifically to CD44-positive cancer cells. Among natural polymers, hyaluronic acid is perhaps the closest approximation to an “ideal” targeting polymer because it combines carrier functionality with intrinsic receptor recognition. Nevertheless, heterogeneity of CD44 expression between patients remains a major obstacle to consistent clinical performance [24,25,26,27,28].

ALG is an anionic polysaccharide that consists of β-D-mannuronic acid and α-L-guluronic acid (G) units. ALG -based PTX systems include NPs, microspheres, hydrogels, and nanofibers. Particularly attractive are calcium-crosslinked alginate systems capable of sustained PTX release. Although ALG is highly attractive for localized drug delivery applications, its role in systemic PTX delivery remains comparatively limited due to stability concerns [1,2,4].

Dex is a branched glucan polysaccharide produced by bacterial fermentation. It consists primarily of α-(1→6)-linked D-glucose units with varying degrees of branching. Dex has been utilized to produce PTX-nano-DDSs (NPs, nanogels, and polymer-drug conjugates). Dex offers excellent safety characteristics but often requires chemical modification before becoming suitable for hydrophobic PTX delivery. Although Dex is highly biocompatible, its hydrophilic nature limits efficient encapsulation of PTX. To address this limitation, numerous derivatives have been developed, such as acetylated Dex, cholesterol-modified Dex, alkylated dextran, and PEG-Dex copolymers. Modified Dex occupy an intermediate position between synthetic and natural polymers and may represent attractive candidates for translational development due to their established biomedical use [1,2,4].

CE is generally unsuitable for PTX delivery due to poor solubility. Consequently, derivatives are utilized in DDS technology (carboxymethyl CE, hydroxypropyl CE, and CE acetate). These materials have been employed in NPs, nanofibers, and hydrogels [1,2].

CDs are cyclic oligosaccharides composed of glucose units. The most common forms include α-CD, β-CD, and γ-CD. Their unique structure contains a hydrophilic exterior and a hydrophobic cavity. This architecture enables the formation of inclusion complexes with hydrophobic PTX. CD-based DDSs improve solubility, stability, and bioavailability. Many formulations combine cyclodextrins with other polymers to generate multifunctional NCs [1,6,7].

Summarizing, natural polymers offer clear biological advantages over many synthetic materials. HA provides intrinsic targeting capabilities, CS enhances cellular uptake, and CD markedly improves PTX solubility. However, from a translational perspective, their greatest weakness remains variability. Unlike synthetic polymers, natural materials frequently exhibit source-dependent differences in molecular weight, purity, regular structure, and biodegradation behavior.

Consequently, many of the most promising PTX nano-DDSs increasingly combine natural polymers with synthetic materials, seeking to balance biological functionality with pharmaceutical reproducibility.

### 3.3. Polymeric Nanoparticles

Polymeric NPs represent the most extensively studied category of PTX-DDSs (Table 1). These structures generally consist of solid colloidal particles ranging from approximately 10 to 300 nm in diameter and may be classified as nanospheres or nanocapsules depending on their internal architecture [4,33,45,46,55]. In nanospheres, PTX is uniformly distributed throughout the polymer matrix, whereas nanocapsules contain a distinct core–shell structure in which the drug is confined within a central cavity surrounded by a polymeric membrane. The choice between these architectures significantly influences drug loading efficiency and release kinetics. Commonly employed polymers include: poly(lactic acid)/polylactide (PLA), poly(glycolic acid) (PGA), glycolide and lactide copolymers (PLGA), polycaprolactone (PCL), polyethylene glycol (PEG), and PEG-containing copolymers. These materials exhibit favorable biodegradability and have established safety profiles, facilitating potential clinical translation. Polymeric nanoparticles offer several advantages for PTX delivery, including enhanced aqueous dispersibility, protection against enzymatic degradation, controlled drug release, improved pharmacokinetics, and passive and active tumor targeting capabilities. However, challenges remain regarding large-scale manufacturing, reproducibility, long-term stability, and regulatory approval [1,45,46,55].

A broad range of polymeric NP platforms has been developed for PTX delivery. The most extensively investigated materials include glycolide and lactide copolymers (PLGA), polylactides (PLA), polyethylene glycol (PEG), PEG-PLGA, PEG-poly(ε-caprolactone) (PCL), PCL, dendritic polymers, and polymer-drug conjugates. These NPs can encapsulate PTX within a biodegradable polymer matrix and provide controlled release, prolonged circulation, protection against degradation, active targeting opportunities, and co-delivery of therapeutic agents. Surface functionalization with ligands such as folic acid, hyaluronic acid, antibodies, aptamers, peptides, and transferrin has further expanded their capabilities [34,48,49,53]. Several preclinical studies have demonstrated remarkable improvements in tumor accumulation and therapeutic efficacy. However, relatively few polymeric PTX NPs have progressed beyond early clinical evaluation [4,12].

Although polymeric NPs represent the most extensively investigated platform for PTX delivery, their superiority over other polymeric systems cannot be considered universal. Their principal advantages include high formulation versatility, the possibility of surface functionalization, and the ability to co-deliver multiple therapeutic agents. Nevertheless, these systems still suffer from relatively low drug loading for certain hydrophobic drugs, premature drug leakage during circulation, and considerable batch-to-batch variability resulting from complex fabrication procedures. Another important limitation is the frequent reliance on the EPR effect, whose clinical relevance in human tumors remains highly heterogeneous. Furthermore, many promising formulations demonstrate excellent efficacy in murine xenograft models but fail to reproduce these results in more clinically relevant models. Consequently, future studies should focus not only on improving targeting efficiency but also on developing scalable manufacturing processes, standardized quality-control procedures, and robust translational strategies to facilitate regulatory approval.

### 3.4. Polymeric Micelles

Polymeric micelles are self-assembled nanostructures formed from amphiphilic block copolymers (Table 1). They typically have diameters of 10–100 nm and consist of a hydrophobic core surrounded by a hydrophilic shell [15,16,23]. The hydrophobic core provides an ideal environment for encapsulating poorly water-soluble PTX, while the hydrophilic corona stabilizes the system in aqueous media and prolongs systemic circulation. This architecture enables substantial improvements in PTX solubility without requiring toxic surfactants such as Cremophor EL [6,15]. Frequently used block copolymers include: PEG-PLA, PEG-PCL, PEG-PLGA, PEG-poly(aspartic acid), PEG-poly(amino acid) derivatives. Polymeric micelles are particularly attractive because of their high drug-loading efficiency, excellent biocompatibility, prolonged circulation, and relatively straightforward manufacturing processes. Nevertheless, micellar systems may undergo premature dissociation upon dilution in the bloodstream, potentially leading to drug leakage and reduced therapeutic efficacy [16,23,26,28,35,36,52].

Genexol-PM^®^ represents one of the first clinically approved polymeric nanomedicines containing PTX (micelle DDSs). The formulation utilizes biodegradable methoxy polyethylene glycol–poly(D,L-lactide) (mPEG-PDLLA) block copolymers that self-assemble into polymeric micelles capable of encapsulating hydrophobic PTX [6,15,36,52]. Polymeric micelles offer several advantages: increased PTX solubility, elimination of Cremophor EL, prolonged circulation, improved drug stability, and enhanced tumor accumulation. Clinical studies have demonstrated that Genexol-PM^®^ permits administration of substantially higher PTX doses than Taxol^®^ while maintaining an acceptable safety profile. Maximum tolerated doses exceeding those achievable with conventional formulations have been reported. However, despite promising clinical performance, the formulation has not achieved global adoption comparable to Abraxane^®^ [15,36,52].

Paclical^®^ represents another Cremophor-free PTX formulation that utilizes a retinoid-based surfactant system to improve PTX solubilization. Similar approaches have sought to eliminate toxic excipients while maintaining pharmacological efficacy. Numerous additional polymeric micelle systems have entered clinical evaluation, including: NK105, Nanoxel^®^, Cynviloq™, and PM-Paclitaxel formulations. Many of these systems demonstrate improved tolerability and favorable pharmacokinetic characteristics. Nevertheless, translation into routine clinical practice has often been hindered by manufacturing challenges, economic considerations, and insufficient superiority over established therapies [2,3,4,6,15].

Polymeric micelles are particularly attractive for the delivery of hydrophobic drugs such as PTX because of their simple self-assembly and excellent solubilization capacity. However, their major weakness remains thermodynamic instability after dilution below the critical micelle concentration following intravenous administration. This instability may lead to premature drug release before reaching the tumor site, thereby reducing therapeutic efficacy. Although cross-linked and unimolecular micelles significantly improve structural stability, they also increase synthetic complexity and manufacturing costs. Moreover, active targeting ligands frequently enhance cellular uptake in vitro, whereas their contribution to therapeutic benefit in vivo remains less evident. Therefore, balancing colloidal stability, PTX release kinetics, and manufacturing simplicity remains one of the major challenges limiting broader clinical translation of polymeric micelles.

### 3.5. Nanogels

Nanogels are three-dimensional crosslinked polymer networks capable of absorbing substantial quantities of water while maintaining structural integrity. Their highly hydrated architecture resembles biological tissues and contributes to excellent biocompatibility [15,26,31].

For PTX delivery, nanogels offer several unique advantages: high drug-loading efficiency, stimuli-responsive release, prolonged circulation, and reduced protein adsorption. Particularly attractive are pH-sensitive nanogels designed to exploit the acidic tumor microenvironment. Such systems remain relatively stable under physiological conditions but rapidly release PTX following exposure to lower pH values characteristic of tumor tissues and intracellular endosomal compartments. Although highly promising, nanogels remain largely confined to preclinical development due to manufacturing and scalability challenges [1,26,31].

Injectable and implantable polymeric hydrogels represent one of the most promising strategies for localized PTX delivery, particularly following surgical tumor resection. Their ability to provide sustained PTX release over prolonged periods while minimizing systemic toxicity constitutes a major therapeutic advantage. Nevertheless, hydrogels are generally unsuitable for treating disseminated metastatic disease because drug diffusion is limited to the site of local administration. Moreover, polymer degradation rates, mechanical properties, and drug release kinetics are strongly influenced by the local physiological environment, making therapeutic performance difficult to predict. Sterilization, storage stability, and reproducible gel formation further complicate clinical implementation. Consequently, optimization of biodegradable hydrogel formulations should be accompanied by standardized manufacturing and quality control protocols.

### 3.6. Star Polymers and Dendrimers

Dendrimers are highly branched synthetic macromolecules characterized by monodisperse structures and precisely controlled architectures. Their unique topology consists of a central core, repeated branching units, and multiple terminal functional groups. This structure provides exceptionally high surface functionality and enables conjugation of drugs, imaging agents, targeting ligands, and therapeutic biomolecules [15,31,32]. PTX may be physically encapsulated within dendritic cavities, covalently attached to terminal groups, and incorporated through polymer-drug conjugation strategies. Poly(amidoamine) (PAMAM) dendrimers are among the most extensively investigated classes for PTX delivery. The major limitation of dendrimers is the potential toxicity associated with their positively charged terminal groups, which may induce membrane disruption and hemolysis. Consequently, extensive surface modification is often necessary before clinical application [15].

Dendrimers possess exceptionally well-defined architectures, high PTX-loading capacity, and numerous functional groups that facilitate precise surface modification. These characteristics make them highly attractive multifunctional nanocarriers for targeted PTX delivery. Nevertheless, higher-generation dendrimers often exhibit increased cytotoxicity associated with positively charged terminal groups, necessitating additional surface modifications such as PEGylation or acetylation. Such modifications improve biocompatibility but simultaneously increase synthetic complexity and production costs. Moreover, relatively limited clinical experience with dendrimer-based drug delivery systems continues to impede regulatory acceptance. Consequently, despite their remarkable structural advantages, further studies addressing long-term safety and large-scale production are required before widespread clinical implementation becomes feasible.

### 3.7. Polymeric Nanofibers

Nanofibers have recently emerged as an innovative platform for localized PTX delivery. Nanofibers are typically implanted directly at tumor sites or surgical resection margins, enabling sustained local drug release over extended periods [18,19,30,37,43,44,47,51,54,56,59,62,63,64]. Electrospinning remains the most widely employed fabrication technique. Nanofibers may be produced from PLA, PLGA, PCL, chitosan, gelatin, and hybrid polymer blends.

Their principal advantages include extremely high drug-loading efficiency, localized delivery, reduced systemic toxicity, and prolonged release kinetics. Nanofiber-based systems are particularly attractive for preventing local tumor recurrence following surgical resection. However, relatively few studies have progressed beyond preclinical evaluation [18,19,51,54,62].

Polymeric nanofibers provide unique opportunities for localized chemotherapy owing to their exceptionally high surface area, prolonged PTX release, and ability to support tissue regeneration simultaneously. These characteristics make them particularly attractive for post-surgical treatment aimed at preventing local tumor recurrence. However, unlike intravenously administered nanocarriers, nanofibers are primarily applicable to localized malignancies and therefore possess limited utility for metastatic disease. Furthermore, reproducible large-scale fabrication of electrospun nanofibers with consistent morphology and drug distribution remains technically demanding. The relationship between fiber architecture, polymer composition, and drug release kinetics is highly system-dependent, making direct comparisons between studies difficult. Future research should therefore focus on establishing standardized manufacturing protocols and validating therapeutic efficacy in clinically relevant postoperative cancer models.

### 3.8. Comparative Analysis of Polymeric Platforms

The remarkable diversity of polymeric DDSs developed for PTX over the past two decades clearly demonstrates that no single nanocarrier platform can simultaneously satisfy all the physicochemical, biological, technological, and regulatory requirements necessary for successful clinical translation. Instead, each polymeric platform offers a distinct balance among drug-loading capacity, release characteristics, targeting potential, manufacturing complexity, and translational feasibility. Consequently, selecting the most appropriate delivery system should be guided not only by PTX properties but also by tumor biology, route of administration, intended therapeutic objective, and clinical setting.

Polymeric NPs remain the most extensively investigated platform because of their versatility, relatively straightforward fabrication, and broad compatibility with both synthetic and natural polymers. Their physicochemical properties can be readily adjusted by controlling particle size, surface charge, polymer composition, and surface functionalization, allowing efficient encapsulation of PTX and incorporation of active targeting ligands. However, despite encouraging preclinical results, many nanoparticle formulations still rely heavily on the EPR effect, whose clinical relevance has proven considerably less predictable than initially anticipated. Tumor vascular heterogeneity, elevated interstitial fluid pressure, dense extracellular matrix, and patient-to-patient variability substantially reduce nanoparticle accumulation within human tumors compared with experimental animal models. Consequently, optimization of NPs design alone is unlikely to overcome the biological barriers that limit drug delivery in clinical oncology.

Polymeric micelles provide excellent solubilization of hydrophobic molecules such as PTX and generally exhibit simpler preparation procedures than many other polymeric systems. Nevertheless, their therapeutic performance remains highly dependent on structural stability after intravenous administration. Conventional micelles may rapidly dissociate upon dilution below their critical micelle concentration, leading to premature drug release and reduced tumor accumulation. Although cross-linked and unimolecular micelles have largely addressed these limitations, they require increasingly sophisticated polymer synthesis, which can complicate regulatory approval and large-scale manufacturing. Thus, improvements in biological performance are frequently accompanied by increased technological complexity.

Polymer-drug conjugates represent an attractive alternative because the covalent attachment of PTX minimizes premature drug leakage and enables precise control of release kinetics through rational linker design. However, selecting an appropriate linker chemistry remains particularly challenging, as excessive stability can prevent efficient intracellular drug release, whereas insufficient stability can lead to premature cleavage in circulation. Furthermore, reproducible synthesis, purification, and characterization of polymer-drug conjugates are substantially more demanding than those of physically encapsulated systems, creating additional manufacturing and regulatory challenges.

Unlike intravenously administered nanocarriers, injectable hydrogels and electrospun nanofibers are primarily intended for localized drug delivery. These systems are particularly attractive for preventing postoperative tumor recurrence because they provide sustained local PTX release while minimizing systemic toxicity. Nevertheless, their clinical application remains largely restricted to localized disease and cannot adequately address disseminated metastases. Moreover, successful implementation requires reproducible control over polymer degradation, drug release kinetics, sterilization procedures, and long-term storage stability, all of which remain significant technological challenges.

Collectively, these observations indicate that future progress in polymeric PTX delivery should extend beyond the continuous development of increasingly sophisticated NCs. Instead, greater emphasis should be placed on overcoming the translational barriers that continue to limit successful clinical implementation. These include reproducible large-scale manufacturing, standardized characterization methods, batch-to-batch consistency, sterilization compatibility, regulatory compliance, long-term safety evaluation, and cost-effective production. Furthermore, future preclinical studies should increasingly employ clinically relevant animal models that better reproduce the biological complexity and heterogeneity of human tumors rather than relying predominantly on rapidly growing xenograft models.

Finally, the future of polymeric PTX nanomedicine will likely depend on the rational integration of material science, pharmaceutical engineering, tumor biology, and translational medicine. Rather than pursuing universal “next-generation” nanocarriers, future research should focus on designing application-specific polymeric platforms optimized for particular clinical indications, routes of administration, and therapeutic objectives. Such an interdisciplinary and clinically oriented approach may ultimately prove more valuable than incremental improvements in carrier complexity alone and could substantially accelerate the translation of polymeric PTX delivery systems from experimental laboratories into routine clinical oncology.

## 4. Mechanisms of Paclitaxel Release from Polymeric Nanosystems and Targeting Nanosystems to Cancer Cells

As is well known, polymeric therapeutic nanosystems have been designed not only to improve PTX solubility and increase its accumulation in tumors, but also to ensure controlled drug release. The release profile is one of the most important parameters determining the therapeutic efficacy of nanosystems, as it influences both the drug concentration at the site of action and the duration of its exposure to cancer cells. Unlike conventional formulations, in which the active ingredient is released almost immediately after administration, polymeric carriers enable gradual, highly controlled, and often selective release of PTX.

The most common mechanism of PTX release from polymeric nanocarriers is diffusion, during which drug molecules move from the interior of the polymer matrix into the surrounding environment along a concentration gradient. This process is particularly dominant in systems based on hydrophobic polymers such as PLGA, PLA, PCL, or PEG-PLA, in which PTX is physically encapsulated within the core of the nanoparticle or micelle. The diffusion rate depends primarily on particle size, matrix porosity, polymer hydrophobicity, and the degree of interaction between PTX and the carrier material. Stronger hydrophobic interactions typically lead to slower release and prolonged therapeutic action.

The second primary mechanism is polymer degradation, which plays a particularly important role in biodegradable polyesters. Hydrolysis of ester bonds leads gradually breaks down of the matrix, releasing previously entrapped PTX molecules. The degradation rate depends on the polymer’s chemical composition, its molar mass, degree of crystallinity, and the ratio of hydrophilic to hydrophobic segments. For example, PLGA nanoparticles with a higher glycolic acid content degrade faster than materials richer in lactic acid, resulting in faster drug release.

In many systems, surface or volume erosion processes occur simultaneously, coexisting with diffusion and degradation. In surface-eroding polymers, PTX release is relatively linear, whereas volumetric degradation often yields a characteristic biphasic profile with an initial burst followed by sustained, controlled release. Limiting the initial burst release is one of the most important challenges in designing polymeric nanosystems, as excessively rapid release of PTX can increase systemic toxicity and shorten the duration of therapeutic action.

Stimulus-responsive systems, which enable selective drug release exclusively within the tumor microenvironment, are also gaining in importance. These systems exploit differences between healthy tissue and tumor, such as reduced pH, elevated glutathione concentration, increased proteolytic enzyme activity, or excessive production of reactive oxygen species. In such nanosystems, carefully designed chemical bonds are broken only upon reaching the tumor, thereby minimizing premature drug release during circulation and increasing therapeutic selectivity. Disulfide bonds, sensitive to reducing environments, and hydrazone or ester bonds, sensitive to acidic pH, are particularly common.

The architecture of the nanosystem also influences PTX release kinetics. In polymer micelles, PTX is typically enclosed in a hydrophobic core, from which it is released primarily by diffusion. In matrix nanoparticles, diffusion and polymer degradation predominate simultaneously, while in polymer-PTX conjugates, the controlled cleavage of the chemical bonds linking the drug to the carrier plays a key role. Hydrogels and electrospun nonwovens, on the other hand, enable localized PTX release lasting several weeks or even months thanks to the slow swelling and degradation of the material. A particularly comprehensive overview of the interrelationships between polymer properties, carrier architecture, and release profile is presented in a recent review on PTX-containing nanofibers.

In summary, the mechanism of PTX release from polymeric nanosystems typically involves a combination of diffusion, polymer degradation, matrix erosion, and processes activated by stimuli specific to the tumor microenvironment. Rational design of these mechanisms allows for optimal pharmacokinetic profiles, increased PTX accumulation in tumors, reduced toxicity to healthy tissues, and improved translational potential of modern polymeric therapeutic systems.

One of the primary objectives of polymeric nanosystems is selective accumulation within tumor tissues while minimizing exposure of healthy organs. As is known, two major targeting strategies are employed: passive and active targeting.

Passive targeting relies primarily on tumor-associated vascular abnormalities and altered lymphatic drainage. Historically, this phenomenon has been described as the Enhanced Permeability and Retention (EPR) effect [1,2,3,4]. Tumor vasculature is frequently characterized by enlarged endothelial gaps, irregular vessel architecture, defective basement membranes, and impaired lymphatic clearance. These features theoretically facilitate preferential accumulation of PTX-NPs within malignant tissues [4,9].

However, recent evidence has challenged the universal applicability of the EPR paradigm. Clinical studies indicate that nanoparticle accumulation in human tumors is highly heterogeneous and often substantially lower than observed in animal models [6,9].

Active targeting involves surface functionalization of NPs with ligands that recognize receptors overexpressed on tumor cells or tumor-associated stromal components [12,15]. Common targeting ligands include folic acid, hyaluronic acid, transferrin, RGD peptides, antibodies, and aptamers. These ligands promote receptor-mediated endocytosis and may significantly enhance intracellular PTX accumulation. Examples particularly relevant to PTX delivery include hyaluronic acid (CD44 targeting), trastuzumab (HER2 targeting), folic acid (Folate receptor targeting), and RGD peptides (Integrin). Numerous preclinical studies have demonstrated superior therapeutic outcomes with actively targeted PTX NPs compared with non-targeted systems. Nevertheless, clinical translation remains limited because receptor expression is often heterogeneous both between patients and within individual tumors [2,3,4,7].

To resume, it should be strongly emphasized that the therapeutic performance of polymeric NCs depends not only on their ability to encapsulate PTX and control its release but also on their capacity to modulate pharmacokinetic behavior and intracellular transport. Consequently, contemporary NCs design increasingly focuses on optimizing systemic circulation, tumor accumulation, cellular internalization, intracellular trafficking, and controlled intracellular PTX release rather than solely improving drug solubility.

One of the principal objectives of polymeric nanosystems is pharmacokinetic modulation. Compared with free PTX, polymeric NCs generally prolong blood circulation, reduce renal clearance, protect the drug from premature degradation, and decrease nonspecific distribution to healthy tissues. Parameters including particle size, surface charge, hydrophilicity, polymer composition, and surface functionalization strongly influence biodistribution and biological interactions. In particular, PEGylation remains one of the most widely employed strategies to reduce protein adsorption, minimize recognition by the mononuclear phagocyte system, and extend systemic circulation. However, recent studies indicate that prolonged circulation alone is insufficient to ensure efficient tumor delivery because nanoparticle accumulation is further limited by tumor heterogeneity, abnormal vasculature, and variability in the EPR effect.

Following tumor accumulation, therapeutic efficacy depends largely on intracellular trafficking. Most polymeric NCs enter cancer cells via receptor-mediated endocytosis following interaction with surface ligands such as hyaluronic acid, folic acid, peptides, antibodies, or aptamers that target overexpressed receptors, including CD44, folate receptor, HER2, EGFR, and integrins. Ligand-mediated internalization substantially enhances cellular uptake while improving selectivity toward malignant cells. After internalization, nanoparticles are transported through early endosomes and subsequently mature into late endosomes and lysosomes, where acidic pH and hydrolytic enzymes may trigger degradation of biodegradable polymers and initiate drug release. Efficient intracellular trafficking, therefore, represents a critical determinant of therapeutic activity because premature lysosomal degradation may reduce cytosolic drug availability. Surface engineering strategies that facilitate endosomal escape, including pH-responsive polymers, proton-sponge materials, membrane-disruptive peptides, or redox-responsive linkers, are increasingly incorporated into advanced NCs to improve intracellular drug delivery.

The intracellular fate of polymeric NCs is equally important for successful therapy. Depending on carrier composition and architecture, polymeric NPs may undergo complete biodegradation into biocompatible metabolites, remain temporarily sequestered within endolysosomal compartments, or release PTX in response to intracellular stimuli such as elevated glutathione concentrations, acidic pH, increased reactive oxygen species, or tumor-associated enzymes. Stimuli-responsive polymeric systems enable selective intracellular drug release while minimizing premature leakage during circulation. Such strategies improve intracellular PTX concentration, reduce systemic toxicity, and may overcome multidrug resistance by bypassing membrane drug efflux pumps through intracellular drug deposition.

Current trends in polymeric nanomedicine therefore emphasize the rational integration of pharmacokinetic optimization, active targeting, control of intracellular trafficking, and stimuli-responsive intracellular drug release within multifunctional NCs. Rather than considering these processes independently, modern nanosystem design increasingly integrates them into a single therapeutic platform that maximizes tumor-selective drug delivery and improves therapeutic efficacy and translational potential. These advanced design principles are expected to play an increasingly important role in the development of next-generation polymeric PTX delivery systems for clinical oncology.

## 5. Problems with Translating Research Results on Paclitaxel Nanosystems

Although remarkable progress has been achieved in the development of polymeric PTX nanosystems, relatively few formulations have successfully advanced to clinical practice. Importantly, successful translation depends not only on superior therapeutic efficacy demonstrated in experimental models but also on the ability to satisfy numerous pharmaceutical, engineering, regulatory, and economic requirements simultaneously. Future nanocarriers should therefore be designed according to a “clinical translation-by-design” strategy, in which manufacturability, reproducibility, scalability, long-term physicochemical stability, sterilization compatibility, and regulatory compliance are considered from the earliest stages of formulation development rather than after proof-of-concept studies. An important observation emerges when reviewing the clinical history of PTX-nano-DDSs.

Compared with intravenously administered nanosystems, implantable PTX delivery platforms such as electrospun nanofibers, biodegradable scaffolds, and polymeric implants offer a fundamentally different therapeutic strategy by delivering PTX directly to the surgical bed after tumor resection. This localized approach enables sustained PTX release over several weeks while minimizing systemic exposure and reducing dose-limiting toxicities. Consequently, implantable systems are particularly attractive for preventing local tumor recurrence, especially in solid tumors, where microscopic residual disease often persists after surgery. Recent reviews identify implantable nanofibers as one of the most promising directions for localized PTX therapy because they combine high drug-loading capacity, prolonged release, mechanical flexibility, and straightforward intraoperative implantation. Despite these advantages, the clinical translation of implantable systems presents regulatory challenges that differ substantially from those of conventional intravenous nanomedicines. Since these products combine a biodegradable biomaterial with a pharmacologically active compound, they are generally considered drug-device combination products and therefore require a comprehensive evaluation of both pharmaceutical and medical device characteristics. In addition to conventional assessments of PTX quality and efficacy, regulatory approval requires demonstration of sterility, mechanical stability, reproducible manufacturing, biocompatibility in accordance with international standards, and detailed characterization of polymer degradation products and their long-term safety. An equally important consideration is the biodegradation profile of implantable polymers. Ideally, the degradation kinetics should closely match the therapeutic window, maintaining controlled PTX release for several weeks before gradually degrading into non-toxic metabolites that can be metabolized or eliminated without inducing chronic inflammation or fibrotic encapsulation. Excessively rapid degradation may result in burst drug release, whereas overly slow degradation may prolong foreign-body responses and delay tissue remodeling. From a surgical perspective, implantable nanofibers and biodegradable scaffolds should be regarded as complementary rather than competing with systemic chemotherapy. Their greatest clinical value lies in immediate placement within the post-resection cavity, where they may eradicate residual microscopic tumor cells, maintain high local PTX concentrations, reduce postoperative recurrence, and potentially decrease the need for repeated systemic chemotherapy. Future clinical implementation will therefore require close collaboration among biomaterials scientists, pharmaceutical developers, surgical oncologists, and regulatory authorities to optimize implant design, establish standardized manufacturing protocols, and generate robust clinical evidence supporting safety and long-term therapeutic benefit.

**Figure 6:**
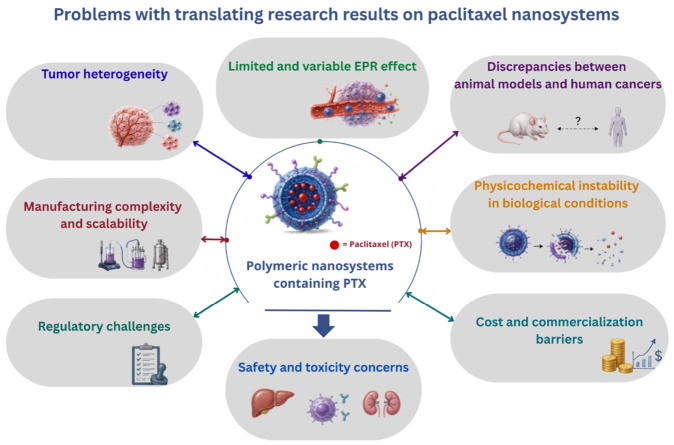
Problems with translating research results on paclitaxel nanosystems. Some graphic elements (mouse, cell, blood vessel, organs, reactors, human) were generated by AI. The manuscript authors independently prepared the figure concept, scheme overall layout, descriptions, and other graphic elements using the graphic design software Canva and Paint.

Despite numerous published studies on promising PTX nanoformulations, only a small number have received regulatory approval. It influences several important factors (Figure 6).

First, many preclinical studies rely on xenograft mouse models that substantially overestimate NPs accumulation within tumors. The EPR effect observed in rodents is frequently less pronounced in human cancers due to differences in vascular architecture, interstitial pressure, and immune responses. An important challenge is the limited predictive value of many preclinical models. Conventional two-dimensional cell cultures and small animal xenograft models frequently overestimate therapeutic efficacy while underestimating the complexity of human tumors, including heterogeneous vascularization, stromal barriers, immune interactions, and interpatient variability. Consequently, greater emphasis should be placed on advanced translational models, including patient-derived organoids, three-dimensional tumor models, patient-derived xenografts, and immunocompetent animal models that more closely recapitulate the biological environment encountered in clinical oncology [2,6,9]. Although the EPR effect has long been regarded as one of the fundamental principles underlying nanoparticle-mediated drug delivery, increasing evidence indicates that its magnitude is highly heterogeneous among patients and considerably less pronounced in human tumors than in rapidly growing murine xenograft models. Quantitative analyses and modern molecular imaging studies have demonstrated substantial interpatient variability in tumor vascular permeability, perfusion, and nanoparticle accumulation, suggesting that passive targeting alone is unlikely to provide consistent therapeutic benefits across all cancer types. Consequently, the predictive value of conventional animal models for estimating nanoparticle delivery in clinical settings remains limited. These observations have stimulated the development of alternative targeting strategies that are less dependent on passive accumulation. Biomimetic nanosystems, particularly nanoparticles coated with erythrocyte, platelet, leukocyte, stem cell, or cancer cell membranes, have emerged as promising platforms that can prolong systemic circulation, evade immune recognition, and enhance interactions with the tumor microenvironment. Likewise, magnetic NPs guided by externally applied magnetic fields represent another attractive approach for increasing local PTX accumulation while reducing systemic exposure. Unlike passive EPR-dependent delivery, these strategies actively direct nanoparticles toward the tumor site and may therefore improve delivery efficiency in poorly vascularized or stromal-rich tumors. Future clinically successful PTX nanosystems will likely combine multiple targeting mechanisms, integrating active ligand-mediated recognition, biomimetic surface engineering, stimuli-responsive drug release, and image-guided patient stratification. Such multimodal approaches may reduce the dependence on the highly variable EPR effect and substantially increase the probability of successful clinical translation.

Although polymeric PTX nanosystems consistently demonstrate impressive therapeutic efficacy in experimental animal models, their clinical benefits have generally been considerably more modest. Numerous preclinical studies summarized in this review report tumor growth inhibition of 70–95% and a prolonged median survival of approximately 30–100% relative to free PTX or untreated controls, depending on the tumor model and treatment protocol. In contrast, the clinical performance of nanomedicines has typically been characterized by incremental rather than transformative improvements. For example, clinically approved formulations such as albumin-bound PTX primarily provide improved response rates, better tolerability, shorter infusion times, and reduced solvent-related toxicity. In contrast, gains in progression-free or overall survival are generally limited and vary substantially across malignancies and treatment regimens. Consequently, remarkable preclinical efficacy should not be interpreted as a direct predictor of clinical success. Instead, future development of polymeric PTX delivery systems should prioritize translationally relevant endpoints—including overall survival, quality of life, long-term safety, and reproducible manufacturing—in addition to conventional measures of tumor regression. This quantitative perspective highlights that successful clinical translation requires not only highly efficient nanocarriers but also realistic preclinical models that more accurately reproduce the biological complexity of human cancers.

Second, increasing formulation complexity often creates manufacturing challenges. Most PTX nano-DDSs are developed at the laboratory scale. Typical preparation methods include nanoprecipitation, emulsification, solvent evaporation, dialysis, and microfluidics. Although effective in small batches, these techniques often become difficult to reproduce at an industrial scale. Multifunctional NPs containing PTX, targeting ligands, imaging agents, and stimuli-responsive elements may demonstrate activity in laboratory performance but prove difficult to reproduce consistently under Good Manufacturing Practice (GMP) conditions [9].

Third, regulatory requirements for nanomedicines remain incompletely harmonized internationally. The absence of universally accepted characterization standards complicates clinical development and approval processes [2,6,9,11,12]. Regulatory agencies require strict control over particle size, size distribution, surface charge, drug loading, release kinetics, sterility, and stability. Even minor manufacturing variations may alter biological behavior. Achieving reproducibility across large production batches remains a major challenge. Long-term storage introduces additional complications, such as aggregation, drug leakage, polymer degradation, and changes in particle size. Moreover, key regulatory questions include: (1) where do NPs accumulate?, (2) how are they eliminated?, (3) what happens after repeated administration?, (4) do degradation products cause toxicity? Regulatory uncertainty remains a major factor slowing translation. Many promising formulations fail during stability testing despite excellent initial performance.

Finally, economic considerations play a critical role. Advanced NCs often require specialized materials, complex manufacturing, and extensive quality control. Consequently, production costs may be substantially higher than those of conventional formulations. Moreover, it is very important to address reimbursement challenges. Healthcare systems increasingly demand evidence of improved survival, reduced toxicity, enhanced quality of life, and cost-effectiveness. Incremental improvements may not justify dramatically increased costs. Many technically successful nanomedicines fail commercially because the economic advantages do not offset manufacturing costs. Even scientifically successful formulations may fail commercially if production costs exceed achievable clinical benefits. Manufacturability is frequently overlooked during academic development. However, clinical success ultimately depends as much on reproducible manufacturing as on therapeutic efficacy.

Finally, future progress will likely depend less on developing increasingly sophisticated nanocarriers and more on identifying formulations that offer a clear, clinically meaningful advantage over existing therapies while remaining sufficiently simple for large-scale manufacturing and regulatory approval. The successful translation of polymeric PTX nanosystems will therefore require integrating nanotechnology, pharmaceutical engineering, tumor biology, regulatory science, and health economics to transform promising laboratory concepts into clinically applicable anticancer therapies.

## 6. Conclusions, Challenges, and Prospects

Despite PTX being one of the most successful chemotherapeutic agents used against breast, ovarian, lung, pancreatic, and several other solid tumors, its clinical utility remains restricted by extremely poor aqueous solubility, dependence on toxic solubilizing excipients, non-specific biodistribution, dose-limiting toxicities, rapid systemic clearance, and the emergence of multidrug resistance.

Polymeric nano-DDSs containing PTX represent one of the most promising directions in modern anticancer drug delivery. The reviewed literature clearly indicates that polymer-based NCs can address several major limitations of conventional PTX therapy. By improving drug solubilization, protecting PTX from premature degradation, prolonging circulation time, enabling controlled release, and enhancing tumor accumulation, polymeric nanosystems may substantially expand the therapeutic index of PTX.

A major advantage of polymeric PTX nanosystems lies in their structural and functional versatility. Biodegradable and biocompatible polymers such as PLGA, PLA, PCL, PEG-based copolymers, CS, HA, ALG, Dex, CD derivatives, and other polysaccharides provide flexible platforms for the design of NPs, micelles, nanogels, hydrogels, macromolecular conjugates, nanofibers, and hybrid systems. These NCs can be engineered to modulate particle size, surface charge, hydrophilicity, degradation rate, PTX-loading capacity, and release kinetics, allowing formulation properties to be adapted to specific tumor types and treatment requirements. Particularly noteworthy is the progress achieved in surface-engineered polymeric nanocarriers. Active targeting strategies have transformed the concept of nanomedicine from passive drug accumulation to more selective, biologically informed delivery approaches. Surface engineering of NCs has emerged as a particularly important strategy for improving the selectivity and efficacy of PTX delivery. Functionalization with ligands such as folic acid, hyaluronic acid, antibodies, peptides, and aptamers enables active targeting of receptors overexpressed on cancer cells, including CD44, HER2, folate receptors, integrins, and other tumor-associated markers. Such receptor-mediated delivery can increase intracellular PTX accumulation, reduce off-target exposure, and improve anticancer activity, especially in aggressive and treatment-resistant tumors.

Another important trend is the development of multifunctional and co-delivery platforms. Since PTX resistance is often driven by multiple mechanisms, including P-glycoprotein-mediated efflux, altered tubulin expression, dysregulated apoptotic signaling, activation of PI3K/Akt, NF-κB, and STAT3 pathways, and protective effects of the tumor microenvironment, single-agent delivery may be insufficient.

Polymeric NCs capable of co-delivering PTX with chemosensitizers, natural compounds, nucleic acids, immunomodulators, phototherapeutic agents, or other cytotoxic drugs offer a rational approach to overcoming resistance and achieving synergistic therapeutic effects.

Polymeric nanofibers and hydrogel-like systems also provide attractive opportunities for localized PTX therapy. Unlike systemic NPs, implantable or injectable polymeric matrices may deliver PTX directly to the tumor bed or post-surgical resection site, maintaining high local drug concentrations while limiting systemic toxicity. This approach is particularly relevant for preventing local recurrence, treating residual disease, and integrating chemotherapy with tissue repair or regenerative strategies.

Despite substantial progress, clinical translation remains limited by several unresolved challenges. Manufacturing complexity, scale-up difficulties, batch-to-batch variability, insufficient reproducibility, and lack of standardized characterization protocols continue to hinder industrial development. Critical quality attributes such as particle size distribution, morphology, PTX-loading efficiency, encapsulation stability, surface ligand density, release profile, sterility, and long-term storage stability must be precisely controlled to meet regulatory expectations.

Biological barriers also remain significant. The enhanced permeability and retention effect is highly variable in human tumors and often fails to produce consistent tumor accumulation. Tumor heterogeneity, dense extracellular matrix, abnormal vasculature, hypoxia, immune clearance, and differences in receptor expression can reduce targeting efficiency and therapeutic predictability. Moreover, the long-term biodistribution, biodegradation, immunogenicity, and potential accumulation of polymeric NCs or their degradation products in non-target tissues require more comprehensive evaluation.

Another major limitation is the persistent gap between preclinical performance and clinical success. Many polymeric PTX nanosystems show excellent results in vitro and in small-animal models, but these outcomes often do not translate into meaningful clinical benefit. Future studies should therefore prioritize clinically relevant tumor models, pharmacokinetic-pharmacodynamic correlations, standardized synergy assessment, biomarker-guided patient selection, and comparative studies against approved PTX formulations such as solvent-based PTX and albumin-bound PTX.

Prospects for the field remain strong. Future polymeric PTX nanosystems should move beyond simple drug encapsulation toward intelligent, adaptive, and clinically translatable platforms. Stimuli-responsive systems activated by pH, redox gradients, enzymes, temperature, light, ultrasound, or magnetic fields may allow more precise control of PTX release. Theranostic nanosystems integrating imaging and therapy could enable real-time monitoring of tumor accumulation, treatment response, and dose optimization. In parallel, advances in precision medicine may support the development of personalized PTX nanotherapies based on tumor receptor profiles, resistance biomarkers, and patient-specific pharmacokinetic characteristics.

In conclusion, polymeric therapeutic nanosystems containing PTX offer a powerful platform for improving the safety, selectivity, and efficacy of PTX-based cancer therapy. Their ability to combine controlled release, active targeting, co-delivery, stimuli-responsive behavior, and multifunctionality makes them highly attractive for next-generation oncology applications. However, successful translation will require not only innovative formulation design but also rigorous standardization, scalable manufacturing, robust safety evaluation, regulatory clarity, and well-designed clinical studies. If these barriers are addressed, polymeric PTX nanosystems may significantly contribute to more effective, personalized, and patient-friendly cancer treatment.

To sum up, the statement that “polymeric nanosystems containing PTX will provide in the future therapeutic efficacy and improve patient safety” is absolutely true.

## Figures and Tables

**Table 1:** Examples of polymeric nanosystems containing paclitaxel.

Polymer(s) Carrier	Nanosystem Type; Properties; Development Stage	PTX System	Advantages	Limitations/Risks	Potential Therapeutic Application	Ref.
Carboxymethyl CS-rhein conjugate	polymeric micelle; <200 nm, DL ≈ 35%; in vitro and in vivo preclinical studies	oral PTX-loaded CR-conjugate micelles	high drug loading (~35%); enhanced intestinal permeation; micelles absorbed as whole;	oral absorption variability; CS derivative batch consistency;	oral colorectal tumor-targeting PTX therapy	[16]
CS-graft-Pluronic F127	aptamer-containing NPs; ≈86 nm, EE ≈ 83%, DL ≈ 9%; in vitro preclinical studies	DNA aptamer-containing CS-graft-Pluronic F127 NPs for PTX	CS biocompatibility plus Pluronic solubilization; aptamer targeting to breast cancer cells;	aptamer stability; Pluronic can affect membrane properties;	breast cancer cell-targeted PTX delivery	[17]
CS/HA	core-sheath nanofiber; ≈300 nm; in vitro preclinical studies	PTX-loaded porous CS fibers coated/immersed in HA	electrostatic CS/HA layering controlled PTX release; HA may add CD44 targeting potential;	natural-polymer variability; mechanical stability and sterilization need optimization;	local cancer chemotherapy with controlled release	[18]
CS core + PCL/CS shell + liposomes	core–shell electrospun nanofiber with liposomal PTX; 70–200 nm; in vitro and in vivo preclinical studies	three-stage release from liposomes, core, shell, and medium; mucoadhesive/biocompatible CS;	complex manufacture; stability of liposomes inside fibers;	multi-stage local chemotherapy	local cancer chemotherapy with controlled release	[19]
Dual-responsive peptide-functionalized NPs	peptide-functionalized NPs; ≈275 nm, EE ≈ 74%, DL ≈ 14%; in vitro and in vivo preclinical studies	peptide functionalized dual-responsive NPs for controlled PTX release	controlled release; enhanced apoptosis in breast cancer cells; ligand-mediated uptake;	peptide stability/proteolysis; synthesis complexity	breast cancer targeted chemotherapy	[20]
Folic acid-grafted PHB + PEG/PVA	amphiphilic polymeric NPs; <200 nm, EE ≈ 79%, DL ≈ 6%; in vitro preclinical studies	PTX-loaded NPs	pH-sensitive release, folate targeting, high EE (~79%), enhanced uptake/apoptosis	mainly in vitro evidence; receptor-expression dependence	breast and colorectal cancer targeted therapy	[21]
Folate-modified lipid-polymer hybrid	hybrid NPs; ≈280 nm, EE ≈ 91%, DL ≈ 27%; in vitro and in vivo preclinical studies	folate-modified lipid-polymer hybrid NPs for targeted PTX delivery	combines polymeric core stability with lipid shell; folate targeting; improved cellular uptake;	hybrid-system manufacturing and stability; off-target folate uptake possible;	targeted cancer chemotherapy	[22]
Gallic acid-CS-TPGS	Polymeric micelle; ≈135 nm; in vitro and in vivo preclinical studies	PTX micelles from gallic acid-CS-TPGS	bioadhesion; P-gp efflux/metabolism inhibition; improved solubility and bioavailability; antitumor effect vs. Taxol in mice;	complex conjugate; surfactant-like TPGS toxicity/PK considerations;	oral/systemic anticancer PTX delivery	[23]
HA	redox-responsive polymeric PTX conjugate; <200 nm; in vitro and in vivo preclinical studies	PTX conjugated to redox-responsive HA polymer	CD44-mediated intracellular delivery; redox-triggered release; improved antitumor effects;	polymer-drug conjugate chemistry complexity; release depends on intracellular redox state	targeted tumor chemotherapy	[24]
HA NPs	HA NPs; 200–300 nm; in vitro and in vivo preclinical studies	HA NPs for targeted PTX therapy	CD44-targeting; natural polymer; in vitro and in vivo antitumor activity;	HA molecular weight and CD44 heterogeneity affect performance	targeted cancer therapy	[25]
HA targeted stimuli-sensitive polymeric nanomicelles	Nanomicelles; 142.5–256 nm, EE ≈ 97%, DL = 7–11%; in vitro preclinical studies	co-encapsulation of PTX and ritonavir to overcome MDR	HA targeting; stimuli-sensitive release; ritonavir can inhibit resistance mechanisms;	drug–drug interaction risk; micelle stability in circulation;	metastatic breast cancer and TNBC MDR therapy	[26]
HA lipoid-coated hybrid NPs	hybrid NPs; <200 nm; in vitro and in vivo preclinical studies	HA-coated hybrid NPs for co-delivery of PTX and curcumin	CD44-targeting potential; synergistic elimination of breast cancer stem cells; co-delivery;	complex co-loading and drug ratio control; HA-CD44 heterogeneity;	breast cancer stem-cell targeting	[27]
Oligo-HA with disulfide linker	GSH-responsive micelles; ≈127 nm, zeta potential = −9 mV, EE ≈ 82%, DL ≈ 21%; in vitro and in vivo preclinical studies	HA-ss-PTX prodrug + ADM co-loaded micelles	CD44 targeting; GSH-responsive release; high drug loading; enhanced blood stability and tumor uptake	dependent on CD44 expression and intracellular GSH; synthesis complexity; preclinical validation only;	CD44-positive cancers (lung and breast cancer models), combination chemotherapy	[28]
Human serum albumin	albumin-bound NPs; ≈127 nm; phase I-II clinical trials	nab-PTX (Abraxane), albumin-bound PTX aggregates	eliminates Cremophor EL; shorter infusion; clinically validated for metastatic breast, NSCLC, pancreatic cancer;	albumin dependence/cost; PTX-albumin binding limits loading; intrinsic PTX toxicities persist	clinically approved systemic chemotherapy	[29]
Lignin NPs + PVA/PVP	composite nanofibrous membrane; <200 nm; in vitro preclinical studies	PTX loaded into lignin NPs, then embedded in PVA/PVP membrane	lignin can improve PTX loading/retention; PVA/PVP membrane improves handling;	potential variability of lignin; multi-step production;	sustained local anticancer delivery	[30]
Branched-PEG	enzyme-responsive nanoassembly; 79–90 nm, DL ≈ 8%; in vitro and in vivo preclinical studies	PTX + capivasertib	Cathepsin B-triggered release, synergistic chemo/AKT inhibition	complex synthesis; dependent on enzyme expression	gastric cancer	[31]
4-arm PEG + riboflavin	targeted polymer-drug conjugate; 122–126 nm; in vitro and in vivo preclinical studies	PEG-PTX-RF	reduced systemic toxicity, active riboflavin targeting, sustained effect;	therapeutic efficacy similar to non-targeted DDS in vivo	solid tumors with RF receptor expression	[32]
DSPE-PEG2000	NPs; <200 nm; in vitro and in vivo preclinical studies	PTX–acrylic acid prodrug (cysteine-responsive)	cysteine-triggered PTX release; GSH depletion; ferroptosis + chemo-immunotherapy synergy; reduced systemic toxicity	requires high intracellular cysteine; complex immune/ferroptosis response may vary; preclinical stage	breast cancer therapy (4T1 model), combined chemo-immuno-ferroptosis treatment	[33]
MIP-PEG-folate	folate-targeted NPs; ≈181 nm, EE ≈ 100%, DL ≈ 16%; in vitro and preclinical studies	PTX MIP-PEG-folate NPs	folate receptor targeting; molecular imprinting can increase PTX recognition/loading;	potential template leakage; synthetic complexity; folate receptor heterogeneity;	folate-receptor-positive cancer therapy	[34]
mPEG-HA-deoxycholic acid-N-acetyl-L-cysteine	redox-sensitive polymeric micelle; ≈147 nm, zeta potential = −38 mV, EE ≈ 74%, DL ≈ 16%; in vitro and in vivo preclinical studies	PTX-loaded redox-sensitive HA graft micelles	EE 73.8%, DL 15.6%, ~147 nm; biocompatible; in vitro and in vivo antitumor effect;	complex graft synthesis; redox trigger may vary between tumors;	HA/CD44-oriented tumor chemotherapy	[35]
mPEG-PDLLA	polymeric micelle; 20–50 nm; phase I clinical trial	Genexol-PM, Cremophor-free polymeric micelle PTX	solvent-free IV formulation; clinically used/approved in Korea; improved tolerability vs. Cremophor formulation;	clinical availability varies by region	metastatic/recurrent breast cancer and NSCLC	[36]
PCL	electrospun membrane/nanofiber; <200 nm, DL ≈ 100%; in vitro and in vivo preclinical studies	pH-responsive PTX release from PCL electrospun membranes	biocompatible, slow-degrading matrix; higher release under acidic pH may match TME;	very hydrophobic; slow or incomplete release at physiological pH;	local cancer therapy where acidic TME triggers faster release	[37]
PCL/Gelatin	tri-layered electrospun nanofiber; <200 nm, DL = 85–97%; in vitro preclinical studies	PCL-gelatin outer layers with central PCL/PTX/5-FU/gelatin reservoir	sequential/local co-chemotherapy; barrier layers can regulate release;	complex architecture; reproducibility and “burst release” control must be verified;	breast cancer local chemotherapy and combination treatment	[38]
PEG-polyaspartate block copolymer	polymeric micelle; 20–240 nm, DL ≈ 23%; in vitro and in vivo preclinical studies	NK105 PTX-encapsulating micelles, ~85 nm	higher tumor/plasma exposure; reduced peripheral nerve distribution; less grade ≥ 3 neuropathy vs. Taxol in Phase II summary;	neutropenia dose-limiting; comparable rather than superior efficacy in the reviewed trial;	advanced/recurrent breast cancer and malignant tumors	[39]
PEGylated liposomes	prodrug-loaded PEGylated liposomes; ≈78 nm, zeta potential = −18 mV, EE ≈ 98%, DL ≈ 7%; in vitro and in vivo preclinical studies	PTX-PA-Liposomes	enhanced stability, longer circulation, higher tumor accumulation	efficacy influenced by esterase activity/immunity	tumor-targeted chemotherapy	[40]
PEGylated phospholipid liposomes	microfluidic PEGylated liposomes; ≈200 nm, EE > 90%; no biological tests	PTX-loaded liposomes	high EE (>90%), size control, good stability	limited active targeting	general anticancer drug delivery	[41]
Peptide-conjugated NPs	tumor-neovasculature-targeted NPs; ≈150 nm, zeta potential = −20 mV; in vitro and in vivo preclinical studies	peptide-conjugated biodegradable NPs carrying PTX	targets tumor neovasculature; improves local accumulation and antiangiogenic effect	peptide–target expression heterogeneity; immunogenicity/proteolysis potential;	antiangiogenic tumor-targeted chemotherapy	[42]
PLA	Electrospun nanofibers; 360–430 nm; in vitro preclinical studies	PLA/PTX nanofibers	simple electrospinning; biodegradable; suitable for localized cancer therapy;	hydrophobic PTX can show incomplete/slow release; residual solvent must be controlled;	cancer therapeutics; local implantable delivery;	[43]
PLA	electrospun nanofibers; 129–445 nm; in vitro preclinical studies	PTX-loaded PLA nanofibers with acidic pH-accelerated release	biodegradable; pH-sensitive release behavior without complex targeting ligand;	limited release at physiological pH; local delivery only;	acidic TME -triggered local chemotherapy	[44]
PLL and PLL/PGA polyelectrolytes	layer-by-layer NCs; 71–120 nm, EE ≈ 100%; in vitro and preclinical studies	PTX-loaded NCs	selective uptake, apoptosis induction, and genotoxic activity in cancer cells	endocytosis-pathway dependence; preclinical stage	breast cancer	[45]
PLL/PGA polyelectrolyte nanocapsules	multicore polyelectrolyte NCs; 90–108 nm, zeta potential = −34 mV, EE ≈ 100%; in vitro preclinical studies	PTX-loaded NCs	reduced cardiotoxicity, mitochondrial protection, and high encapsulation	need further in vivo validation	cancer therapy with improved cardiac safety	[46]
PLA/PEG	Nanofiber mats; 200–600 nm, EE ≈ 100%, DL = 9–11%; in vitro and in vivo preclinical studies	PTX-eluting PLA/PEG mats	PEG increases hydrophilicity and boosts PTX release; antiangiogenic and recurrence-prevention potential;	PEG content can alter mechanical properties and “burst release”; implant-only use;	tumor recurrence prevention after surgery	[47]
PLGA	aptamer-functionalized NPs; 127–143 nm, EE = 31–32%, DL = 3–4%; in vitro and in vivo preclinical studies	HPA aptamer-functionalized PTX-loaded PLGA NPs	active targeting and microenvironment modulation; PLGA biodegradability;	aptamer stability, cost, and scale-up; target heterogeneity;	breast cancer targeted therapy	[48]
PLGA NPs for salinomycin + PTX, CD44-targeted	PLGA NPs; <150 nm; in vitro preclinical studies	co-delivery of salinomycin and PTX for cancer cells plus cancer stem cells	co-eradication concept; CD44 targeting; may overcome resistance;	co-drug toxicity; scaling and ratio control; target heterogeneity;	breast cancer cells and cancer stem cells	[49]
PLGA core -PEG/lipid shell + FA	lipid-polymer hybrid nanocapsules; <200 nm; in vitro preclinical studies	PTX or PTX + DOX	folate targeting, controlled release, improved bioavailability	primarily preclinical evaluation	breast cancer	[50]
PLGA	electrospun implantable nanofiber mat; 650–750 nm; in vitro and in vivo preclinical studies	metronomic PTX release from PLGA nanofibers	improved antitumor effect; reduced metastasis; extended survival compared with intraperitoneal PTX in the TNBC model;	requires implantation; release-rate optimization and local tissue compatibility needed	breast cancer local/metronomic chemotherapy	[51]
PLGA + BFA micelles	emulsion-electrospun nanofiber with embedded micelles; <200 nm; in vitro preclinical studies	PTX plus Brefeldin A micelles in PLGA fibers for dual release	sequential/dual release; PTX rapid and BFA gradual release can support combination therapy;	complex drug-ratio control; possible additive toxicity;	combination chemotherapy with programmed release	[52]
PLGA + leukocyte membrane	biomimetic membrane-camouflaged NPs; 149–171 nm, zeta potential = −11 mV; in vitro preclinical studies	PTX + cannabidiol	immune evasion, prolonged release, reduced hemolysis, anti-metastatic effect	complex manufacturing; biological membrane variability;	breast cancer combination therapy	[53]
PLGA/CS/zeolite or PLGA/CS/MOF	electrospun nanofibers; 45–95 nm; in vitro and in vivo preclinical studies	PTX delivery toward prostate cancer cells with a pH-sensitive CS component	pH 5.5 release enhanced vs. pH 7.4; zeolite/MOF can modulate loading and release	inorganic filler safety and long-term fate; reproducibility concerns	targeted/local prostate cancer chemotherapy	[54]
PLGA + magnetic fluid + fluorescent tag	Theranostic NPs; 218 nm, EE = 29–40%; in vitro preclinical studies	aptamer-conjugated PTX and magnetic-fluid-loaded fluorescent PLGA NPs	combines targeting, magnetic/fluorescent imaging, and therapy	multicomponent regulatory complexity; magnetic-fluid safety and clearance;	targeted cancer therapy and imaging	[55]
PNIPAAm/PU	thermo-responsive nanofiber; 230–340 nm; in vitro and in vivo preclinical studies	PTX release from PNIPAAm/PU nanofibers triggered at elevated temperature	on-demand thermal release; potential synergy with hyperthermia;	external heat required;	hyperthermia-assisted local chemotherapy	[56]
Polyglutamic acid	macromolecular conjugate; <100 nm; phase I and II clinical trials	PTX conjugated to poly-L-glutamic acid)	prolonged half-life and tumor-selective accumulation; reduced neuropathy/neutropenia vs. Taxol;	variable clinical benefit; polymer-drug release depends on tumor enzymes;	systemic chemotherapy for NSCLC/ovarian/breast cancer studies	[57]
Polymeric (PEO-*b*-PCL or PEO-*b*-PBCL) micelles functionalized with cancer-specific peptide ligands	peptide–functionalized polymeric micelles; 50–150 nm; in vitro preclinical studies	cancer-specific peptide–ligand micelles loaded with PTX	active targeting; solubilization of hydrophobic PTX; micellar nanosize;	micelle dissociation/dilution in blood; peptide stability;	active tumor-targeted PTX delivery	[58]
PU/PCL	coated membrane/nanofiber barrier; <200 nm, DL = 3%; preclinical studies	PCL-coated PTX-loaded PU membrane	PCL barrier reduced early burst release (about 20% vs. 40% at 24 h)	complex manufacturing; PU biodegradation/biocompatibility considerations	localized postoperative chemotherapy	[59]

## Data Availability

No new data were created or analyzed in this study. Data sharing is not applicable to this article.

## Abbreviations

The following abbreviations are used in this manuscript:

ADM	adriamycin
ALG	alginate
BFA	Brefeldin A
CD	cyclodextrin
CE	cellulose
CL	ε-caprolactone
CMCS	carboxymethyl chitosan
CS	chitosan
DL	drug loading
DDSs	drug delivery systems
Dex	dextran
DOX	doxorubici
DSPE	diacylphosphatidylethanolamine
EE	encapsulation efficiency
EPR	enhanced permeability and retention effect
FA	folic acid
FDA	United States Food and Drug Administration
5-FU	5-fluorouracil
GA	glycolide
GMP	Good Manufacturing Practice
GSH	glutathione
HA	hyaluronic acid
LA	lactide
MDR	multidrug resistance
MIP	molecularly imprinted polymer
MOF	metal–organic frameworks
mPEG-PDLLA	methoxy polyethylene glycol-poly(D,L-lactide)
nab-PTX	nab-paclitaxel
nano-DDSs	nano drug delivery systems
NC/NCs	nanocarrier/nanocarriers
NP/NPs	nanoparticle/nanoparticles
NSCLC	non-small cell lung cancer
PA	palmitic acid
PAMAM	poly(amidoamine)
PCL	poly(ε-caprolactone)
PGA	poly(glycolic acid)
PEG	polyethylene glycol
PEO-*b*-PCL	poly(ethylene oxide)-*b*-poly(ε-caprolactone) block copolymers
PEO-*b*-PBCL	poly(ethylene oxide)-*b*-poly(α-benzyl carboxylate-ε-caprolactone) block copolymers
PHB	poly-3-hydroxybutyrate
PLA	poly(lactic acid)/polylactides
PLLA	poly(L-lactide)
PDLA	poly(D-lactide)
PDLLA	poly(D,L-lactide)
PLGA	glycolide and lactide copolymers
PNIPAAm	poly(*N*-isopropylacrylamide)
PPC	poly(propylene carbonate)
PTX	paclitaxel
PU	polyurethane
PVA	poly(vinyl alcohol)
PVP	poly(vinyl pyrrolidone)
TME	tumor-microenvironment
TNBC	triple-negative breast cancer
TPGS	D-ɑ-tocopheryl polyethylene glycol succinate
WHO	World Health Organization
